# Double-blind, placebo-controlled trial of mifepristone on cognition and depression in alcohol dependence

**DOI:** 10.1186/s13063-020-04726-z

**Published:** 2020-09-16

**Authors:** Kim Donoghue, Abigail Rose, Simon Coulton, Rachel Coleman, Joanna Milward, Thomas Philips, Colin Drummond, Hilary Little

**Affiliations:** 1 Addictions Department, National Addiction Centre, Institute of Psychiatry, King’s College London, 4 Windsor Walk, London, SE5 8BB UK; 2 Department of Psychological Sciences, University of Liverpool, 2.32, Eleanor Rathbone Building, Bedford Street South, Liverpool, L69 7ZA UK; 3 Centre for Health Service Studies, University of Kent, Canterbury, Kent CT2 7NF UK; 4 Department of Health Sciences, Seebohm Rowntree Building, University of York, Heslington, York, YO10 5DD UK; 5 Institute for Clinical and Applied Health Research, Allam Medical Building,, University of Hull, Hull, HU6 7RX UK

**Keywords:** Alcohol dependence, Memory, Cognitive function, Depression, Cortisol, Glucocorticoid type II receptor, Mifepristone

## Abstract

**Background:**

Alcohol dependence is a significant issue contributing to disease burden. Changes in cortisol concentrations during alcohol withdrawal are associated with cognitive deficits and symptoms of depression. Current treatments are only successful for a small proportion of people and do not target cognitive deficits and symptoms of depression experienced by those who are alcohol dependent. The aim of this research is to determine the potential efficacy of mifepristone, a type II glucocorticoid receptor antagonist, to prevent symptoms of depression and cognitive deficits following alcohol detoxification.

**Methods:**

This was a phase 2 therapeutic use trial. It was a double-blind randomised controlled clinical trial of mifepristone versus inactive placebo treatment. The trial aimed to recruit 120 participants who met the inclusion criteria: (1) male, (2) aged 18–60 years inclusive, and (3) alcohol dependent for 5 or more years. Participants were randomised to 600 mg a day mifepristone (200 mg morning, afternoon, and evening) for 7 days and 400 mg for the subsequent 7 days (200 mg morning and evening) or the equivalent number of placebo tablets for 14 days. Primary outcome measures were cognitive function (measured using the Cambridge Neuropsychological Test Automated Battery (CANTAB)) and symptoms of depression (measured using the Beck Depression Inventory (BDI)) at 4 weeks post-randomisation.

**Results:**

Difficulties recruiting participants due to significant changes in the provision of inpatient care for alcohol dependence resulted in only 27 participants recruited to the trial, with data available for 21 participants. Fourteen participants were randomised to receive mifepristone and 13 to receive placebo.

**Conclusion:**

Larger trials would be needed to draw conclusions about the efficacy of mifepristone.

**Trial registration:**

ISRCTN registry ISRCTN54001953. Registered on 29 September 2011.

## Background

Alcohol dependence is a significant global problem contributing to over 4% of disease burden [1], impacting social, physical, and mental health. Pharmacological and psychosocial treatments for alcohol dependence are only successful for a subsample of people, with up to 70% returning to drinking within the first 12 months following treatment [2]. Recurrent episodes of drinking and withdrawal result in the phenomenon of kindling, with greater severity of withdrawal symptoms including risk of seizure [3] and greater cognitive deficits [4]. New, effective treatments are therefore required to help reduce rates of relapse and the health, social, and economic consequences of multiple episodes of dependent drinking and withdrawal.

Impairment in cognitive function is found in 50 to 80% of those dependent on alcohol with impairment experienced across multiple domains including memory, attention, learning, processing speed, visuospatial abilities, and executive function [5, 6]. These impairments may hinder an individual’s quality of life and their ability to benefit from treatment programmes [7–11]. In addition to cognitive deficits, those with a diagnosis of alcohol dependence are two times more likely to be diagnosed with major depression [12]. Greenfield et al. [13] found that those entering inpatient treatment for alcohol dependence who also had a diagnosis of major depression had a shorter time to first drink and relapse in the first year following treatment.

Alcohol withdrawal-induced abnormalities of the hypothalamic-pituitary-adrenal (HPA) axis function have been associated with both deficits in memory and symptoms of depression [14–16]. High levels of glucocorticoids are released during acute withdrawal from alcohol, and these may have a causative role in the exacerbation of cognitive deficits [17] and persistent glucocorticoid dysregulation following abstinence from alcohol may result in sustained cognitive dysfunction [18]. Furthermore, Errico et al. [19] demonstrated a link between severity of cognitive deficits and the number of withdrawal episodes and higher cortisol levels during acute alcohol withdrawal. Clinical research has demonstrated that a type II glucocorticoid receptor antagonist, mifepristone, has a positive impact on symptoms of depression [20–24] and cognitive function [25].

In preclinical studies, administration of mifepristone early in the acute phase of alcohol withdrawal reduced neuronal toxicity and deficits in cognitive function [26–28]. Sharrett-Field et al. [29] demonstrated that this drug significantly reduced the signs of alcohol withdrawal. Reynolds et al. [30] demonstrated that another type II glucocorticoid antagonist, ORG 34517, reduced the severity of alcohol withdrawal in rats. It was therefore hypothesised that blocking type II glucocorticoid receptors during acute withdrawal could reduce the deficits in cognitive function and symptoms of depression in alcohol dependence. Current pharmacological treatment for alcohol dependence targets alcohol consumption directly and does not tackle the cognitive deficits and symptoms of depression that are experienced following alcohol withdrawal. The current study (MifCog) is the first published report of an investigation examining the potential efficacy of mifepristone given to alcohol-dependent persons during acute alcohol withdrawal to protect against subsequent deficits in cognitive function and symptoms of depression.

## Method

This research aimed to determine the potential efficacy of mifepristone for preventing symptoms of depression and cognitive deficits following alcohol detoxification. It was hypothesised that mifepristone would be associated with greater cognitive function and fewer symptoms of depression in comparison to placebo.

### Participants

Full details of the trial methodology and design are reported in Donoghue et al. [31]. Participants were male (given mifepristone’s antiprogestogen effects, it is unsuitable for female administration), aged between 18 and 60 years old with a diagnosis of alcohol dependence for at least 5 years (determined using the Composite International Diagnostic Interview (CIDI) [32]) and scheduled to complete an alcohol detoxification. Exclusion criteria included the following: a clinical diagnosis of a neuroendocrine disorder, liver damage (alanine aminotransferase (ALT) activity of more than 2.5× normal range), renal dysfunction (creatinine levels over 150 μmol/l in plasma), documented evidence of a psychotic disorder, severe brain damage or severe mental impairment, a diagnosis of severe physical illness that would preclude participation (e.g. terminal illness), documented evidence of current dependence on a substance other than alcohol or nicotine, inability to understand sufficient English to understand the information needed for the cognitive testing, patients with Wernicke-Korsakoff syndromes, porphyria, severe asthma uncontrolled by therapy, a cardiac disorder, persistent high blood pressure (over 160 mmHg systolic and/or 100 mmHg diastolic), a medical history of diabetes, a known allergy to mifepristone, prescription of contraindicated medications, and current participation in another clinical trial.

Participants were identified through community drug and alcohol teams in South East London, Kent, Sussex, Barnsley, and Hull in England. All potential participants were given written and verbal information about the trial before written informed consent was obtained. Medical history was determined by a trained physician through full history, case note, and medication review. Ethical approval was granted by the London–Dulwich Research Ethics Committee (reference: 10/H0808/7) and complies with UK and European Good Clinical Practice for medicinal trials guidelines.

### Primary outcomes

The primary outcomes of this research were depression in the first 4 weeks following initiation of detoxification and cognitive function. Symptoms of depression were assessed on days 7, 14, 21, and 28 using the BDI. Assessment of cognitive function was completed using the Cambridge Automated Test Battery (CANTAB [33]) on days 21 and 28. Specific CANTAB tests were chosen to assess a wide range of domains of cognitive function known to be affected by dependent drinking, which included the following: Motor Screening task that checks for movement and visual problems; Reaction Time task that measures motor skill function; Pattern Recognition Memory; Matching to Sample Visual Search that measures visual matching ability and short-term visual recognition memory; Spatial Recognition Memory; Paired Associates Learning that measures visual memory and new learning; Rapid Visual Information Processing that measures sustained attention; Intra-Extra Dimensional Set Shifting that measures attentional set formation maintenance, shifting, and flexibility; Spatial Working Memory; and One Touch Stockings of Cambridge that measures planning and working memory subdomains of executive function. Cognitive testing did not take place at the initial baseline assessment due to participants being intoxicated; this would have had a significant impact on ability to perform the tests.

### Secondary outcomes

Secondary outcomes collected on days 7, 14, 21 and 28 included the Profile of Mood States (POMS [34]), Snaith-Hamilton Pleasure Scale (SHAPS [35]), previous night’s sleep quality (on a scale of 1–10, with 10 being the best night sleep), and the Alcohol Urge Questionnaire (AUQ [36]). A cut-off score of 2 was used to indicate possible anhedonia on the SHAPS [35]. Urine samples were taken just prior to the cognitive testing on days 21 and 28 after cessation of alcohol drinking to establish the concentrations of the unhydrolysed levels of chlordiazepoxide and the active metabolites norchlordiazepxide (also known as desmethylchlordiazepoxide), oxazepam, demoxepam, and nordiazepam (also known as desmethyldiazepam) [37, 38]. During the initial 4 weeks of the trial, all participants were asked to report any adverse events at each research visit.

### Design and procedure

The study was designed as a double-blind, parallel-group, randomised controlled clinical trial of mifepristone and inactive placebo. It is a phase 2 therapeutic use trial. A baseline assessment was completed a maximum of 4 weeks prior to detoxification to determine eligibility for the trial and to collect data on participant demographics, alcohol consumption (Timeline Follow-back (TLFB) [39], Severity of Alcohol Dependence Questionnaire (SADQ) [40], Alcohol Urge Questionnaire (AUQ) [36]), and symptoms of depression (Beck Depression Inventory (BDI) [41]), see Table 1.

**Table 1:** Participant demographics, drinking-related variables, and depression symptoms at baseline for those randomised and those not randomised

	Randomised (***n*** = 27)	Mifepristone (***n*** = 14)	Placebo (***n*** = 13)	Not randomised (***n*** = 30)
Age, mean (SE)	41.9 (1.6)	42.9 (2.4)	40.8 (2.1)	45.3 (1.7)
White, *n* (%)	26 (96.3)	13 (92.9)	13 (100.0)	18 (85.7)
Married or cohabiting, *n* (%)	2 (7.4)	0 (0.0)	2 (15.4)	1 (4.8)
In employment, *n* (%)	1 (3.7)	0 (0.0)	1 (7.7)	2 (9.5)
Professional or skilled, *n* (%)	9 (33.3)	3 (21.4)	6 (46.2)	10 (47.6)
Home owner, *n* (%)	1 (3.7)	1 (7.1)	0 (0.0)	0 (0.0)
Years of education, mean (SE)	11.1 (0.5)	12.1 (0.6)	10.6 (0.3)	13.1 (0.9)
Number of children, mean (SE)	2.2 (0.3)	2.7 (0.4)	1.9 (0.5)	2.4 (0.3)
Age of first drink, mean (SE)	12.5 (0.5)	13.6 (0.7)	12.6 (0.7)	14.2 (0.5)
Age drinking weekly, mean (SE)	16.4 (0.7)	17.1 (0.7)	17.1 (1.1)	20.0 (2.1)
Age drinking daily, mean (SE)	24.6 (2.7)	26.9 (2.9)	21.4 (1.2)	25.8 (2.81)
Beck Depression Inventory, median (IQR)	33 (13)	32 (9.5)	40 (24.5)	36 (14.5)^1^

^1^Data available for 16 participants

On the first day of detoxification, participants were randomised 1:1 to receive mifepristone or placebo. Randomisation was performed using an online system supported by King’s Clinical Trials Unit at the Institute of Psychiatry, Psychology and Neuroscience. Allocation was stratified by severity of alcohol dependence (SADQ score of 40 or more or under 40), research site, and age (18 to 29, 30 to 39, 40 to 49, or 50 to 60). All participants, clinical staff, and research staff were blind to medication allocation. All tablets were identical, stored in blister packs in cartons labelled with unique ID numbers, which were allocated to participants following randomisation. Participants were administered 200 mg of mifepristone or placebo three times a day (morning, afternoon, evening) for 7 days, starting as soon as possible on the first day of detoxification. This was followed by 200 mg of mifepristone or placebo twice a day (morning and evening) for 7 days.

### Protocol changes

Many potential participants were being excluded from participation in the trial due to a diagnosis of asthma. Clarification of the exclusion criteria for those suffering from asthma was made to exclude only those with severe asthma uncontrolled by therapy. Due to the changes in service provision for the treatment of alcohol dependence and new available research with mifepristone, patients completing their alcohol detoxification as an outpatient were included in the trial. Due to research grant funding limitations, this only included one participant.

### Medication adherence

Supervised dosing of mifepristone/placebo was completed for all participants completing their detoxification as an inpatient for the entire 2-week course of the medication. Those completing their detoxification as an outpatient were asked to record each dose of medication.

### Follow-up assessments

Participants were followed up at 3, 6, and 12 months post-randomisation to assess levels of alcohol consumption (TLFB) and problems associated with alcohol consumption (APQ; 6 and 12 months only), and symptoms of depression, alcohol craving, mood, and anhedonia were collected using the BDI, AUQ, POMS, and SHAPS, respectively.

### Sample size

Previous data on this target population was not available to establish how many participants would be required. However, previously published studies using the CANTAB test battery in depressed patients have shown effect sizes of 0.5, 0.8, 0.76 [42], 0.496, 0.5 [43], 0.897 [44], and, in the aged, 0.7 for spatial working memory [45]. Similar values were obtained studying effects of acute alcohol with an early version of the CANTAB [46]. We estimated the numbers needed in each group for a variety of effect size estimates based on a power of 80% and alpha of 0.05. For an effect size difference of 0.8 25, subjects per group would be required and this increased to 32 for an effect size difference of 0.7 and 63 if the difference was 0.5. We erred on the side of caution and aimed to detect a small to medium effect size of 0.5, and this required 120 subjects participating in the study, 60 in each group.

### Data analysis

Due to under-recruitment of participants, the trial was inadequately powered to perform inferential statistics. Outcomes are therefore presented as means with standard errors (SE) or standard deviations (SD) or medians with interquartile ranges, as appropriate. Mean differences are reported with 95% confidence intervals (CI).

## Results

### Study population and recruitment difficulties

**Fig. 1:**
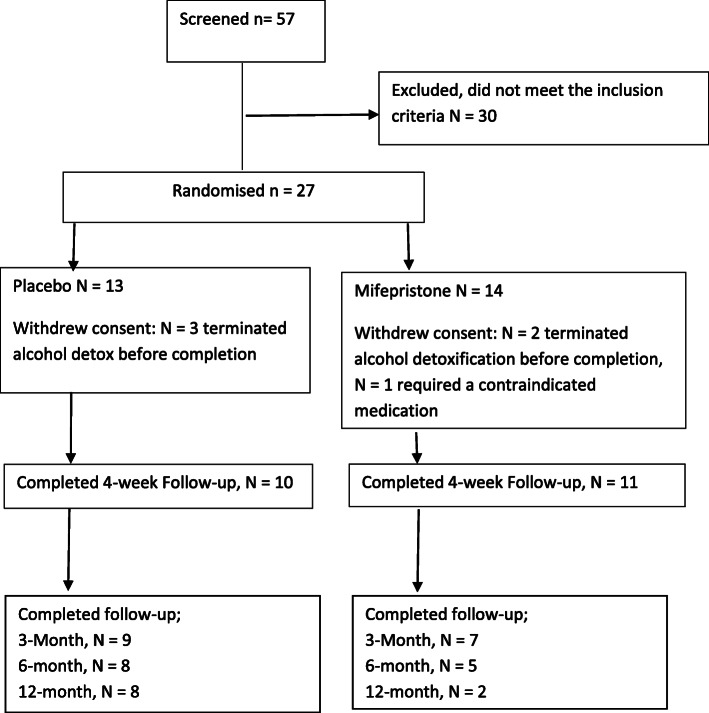
Trial consort diagram

Recruitment for the trial began in September 2012, and the final participant follow-up assessment took place in July 2016. A total of 57 people were screened to take part in the trial, and 27 (47%) were randomised (Fig. 1); Table 1 presents the demographics of these. Similar levels of alcohol consumption, alcohol problems, and craving were apparent for those who were allocated placebo and those who were allocated mifepristone (Table 2). A total of 6 participants withdrew from the trial. Three participants randomised to placebo and two participants randomised to mifepristone withdrew consent owing to terminating their alcohol detoxification and before completion of the trial primary outcome measures. Withdrawal of participants prior to the administration of the primary outcomes resulted in removal of the data for that participant from the study results as per the trial protocol.

**Table 2:** Baseline alcohol consumption characteristics for mifepristone and placebo groups

	Mifepristone (***n*** = 14), median (IQR)	Placebo (***n*** = 13), median (IQR)
Timeline Follow-back, drinks per drinking day	29.5 (18.1)	27.24 (24.3)
Alcohol Problems Questionnaire	13.5 (6.78)	13.0 (6.5)
Alcohol Urge Questionnaire	47.5 (16.78)	51.0 (31.5)

Drink = 1 UK unit equivalent to 8 g/10 ml of pure ethanol

One participant randomised to mifepristone was withdrawn from the trial due to the necessity of treatment with a contraindicated medication. Primary outcome measures were completed for 21 participants.

The study took place at a time of significant policy change in the provision of alcohol services in UK. Commissioners of alcohol services frequently reviewed services and invited new tenders to provide treatment services contracts (i.e. retendering). As a result, there were closures of NHS specialist inpatient detoxification facilities at the participating research sites which provided serious challenges to recruitment, detailed further in the “Discussion” section. The original protocol included only participants who were completing an alcohol detoxification as inpatients. A protocol amendment was made in November 2014 to include participants completing their alcohol detoxification as an outpatient to expand the potential participant pool. However, due to further retendering of alcohol services and funding constraints, the improvement in recruitment was minimal with only one participant recruited as an outpatient.

### Primary outcomes

At 4 weeks post-randomisation, those who received mifepristone had a numerically higher mean BDI score (*n* = 11, mean = 13.77, SE = 2.36) compared to those who received placebo (*n* = 10, mean = 8.50, SE = 2.75) with a mean difference of 5.27 (CI = − 2.86 to 13.40). Table 3 presents the mean (SD) for tests of cognitive function at 4 weeks post-randomisation as well as the mean difference (with 95% confidence intervals) between those receiving mifepristone and those receiving placebo for these scores. A numerically higher score indicates superior performance on the Pattern Recognition Memory, Spatial Recognition Memory, Stockings of Cambridge, and Match to Sample Visual Search tasks, whereas a lower score indicates better performance on all other tasks. Those randomised to receive mifepristone performed better than those randomised to placebo on all tasks of memory (Paired Associate Learning, Pattern Recognition Memory, and Spatial Recognition Memory). However, those randomised to receive placebo performed better than those randomised to receive mifepristone on all tasks of executive function (Stockings of Cambridge, Intra-Extra Dimensional Set Shift, and Spatial Working Memory tasks) and all tasks of attention and psychomotor speed (Reaction Time, Rapid Visual Information Processing, Match to Sample Visual Search).

**Table 3:** Cognitive function at week 4 post-randomisation for mifepristone and placebo groups

Test	Mifepristone (***n*** = 11), mean (SD)	Placebo (***n*** = 10), mean (SD)	Mean difference (95% CI)
**Memory**			
Paired Associates Learning (number of trials)	12.36 (3.47)	12.80 (3.22)	− 0.44 (− 3.70 to 2.83)
Pattern Recognition Memory (% correct)	91.67 (7.45)	90.00 (13.49)	1.67 (− 9.18 to 12.51)
Spatial Recognition Memory (% correct)	73.64 (12.86)	66.50 (17.49)	7.14 (− 8.01 to 22.28)
**Executive Function**			
Stockings of Cambridge (number of problems solved)	7.55 (2.54)	8.10 (1.96)	− 0.55 (− 2.76 to 1.65)
Intra-Extra Dimensional Set Shift (number of trials)	164.82 (115.87)	117.60 (67.00)	47.22 (− 43.57 to 138.01)
Spatial Working Memory (between errors)	53.09 (17.82)	39.00 (21.20)	14.09 (−5.15 to 33.33)
**Attention and psychomotor speed**			
Reaction Time, reaction (milliseconds)	350.44 (66.57)	330.51 (89.82)	19.92 (− 56.59 to 96.44)
Rapid Visual Information Processing latency (milliseconds)	473.69 (114.84)	422.68 (134.90)	51.00 (− 75.16 to 177.17)
Match to Sample Visual Search (% correct)	95.96 (5.60)	96.11 (6.44)	− 0.16 (− 6.08 to 5.77)

### Secondary outcomes

Descriptive statistics are reported for the POMS, SHAPS, AUQ, and sleep quality and are presented in supplementary table 1.

### Follow-up

Descriptive statistics for outcomes at 3-, 6-, and 12-month follow-up are presented in supplementary table 2.

### Adverse events and medication adherence

No participants withdrew from the trial due to medication side effects. One participant randomised to receive placebo experienced a serious adverse event during the trial that was unrelated to the trial medication. There were 54 reported adverse events during the trial (23 placebo, 31 mifepristone); the most common were headache (4 placebo, 4 mifepristone), sickness and nausea (3 placebo, 1 mifepristone), and skin rash and itching (1 placebo, 3 mifepristone). Medication adherence was high with 18 of the 21 participants who completed the 4 weeks of the trial taking 100% of medication; two participants missed one dose and one participant missed two doses of medication.

## Discussion

This was the first RCT aimed to determine the effectiveness of mifepristone in reducing the cognitive deficits and depression symptoms often observed during alcohol detoxification. Due to significant recruitment challenges, analysis only involved 27 participants who met eligibility and were randomised to mifepristone (52%) and placebo (48%). Therefore, no definitive conclusions could be drawn. However, it is worth noting that those who received mifepristone had greater BDI scores at 4 weeks post-randomisation compared to those who received placebo (mean difference = 5.27), indicating greater severity of depression, which may be considered clinically significant [47]. Those randomised to receive mifepristone had scores indicating superior performance on all tasks of memory; however, the opposite was true for all tasks of executive function and attention and psychomotor speed.

Five participants were lost from the trial due to them deciding to discontinue their alcohol detoxification, three had received placebo and two mifepristone, and the sixth because of a contraindicated medication. There were no reported serious side effects related to mifepristone treatment. Compliance was excellent due to medication being administered by medical staff on the inpatient ward for all but one study participant. Three treatment doses in total were missed throughout the study.

### Low participant recruitment rates

It did not prove possible to recruit the number of participants originally aimed for in this study. This was primarily because of repeated recruitment delays due to substantial changes in UK addiction services treatment provision based on new government policy, which is estimated to have resulted in a 44.6% reduction in the number of inpatient treatment admissions over the course of the study [48]. When the trial was started, there were two local inpatient detoxification wards that would have provided many potential participants. These wards were both closed just as the study began. The team then moved to recruiting from other NHS specialist addiction units at participating research sites, with each new centre necessitating substantial paperwork before recruitment could begin. Eight new recruitment centres were established, but four of these were then also lost owing to closures or retendering. In addition, some addiction units changed to only 2 weeks’ inpatient treatment, instead of the 4 weeks that the original wards provided and that the trial protocol required. The trial protocol was amended to allow participants only receiving 2 weeks of inpatient care to take part. As described above, the trial protocol was further amended near the end of the study to include outpatients, but this did not improve the situation. The study then had to cease because the repeated delays resulted in expiration of the funding.

As the study was unable to recruit participant numbers, there was insufficient data to show whether or not mifepristone alleviated or prevented the depressive symptoms and/or cognitive deficits caused by long-term alcohol consumption and withdrawal. It was unfortunate that major changes in government policy on treatment service provision for alcohol dependence, after the commencement of the study and outside the control of the researchers, prevented conclusions from being reached.

### Study limitations

The sample size for this trial was very small owing to difficulties recruiting eligible participants as a result of changes in the inpatient service provision for the treatment of alcohol dependence. Meaningful conclusions could therefore not be drawn. Only males were included in this trial due to mifepristone’s blocking effect on progesterone and the associated risks in women. This limits the generalisability of the trial findings.

### Study strengths

This was the first clinical trial to examine the potential for mifepristone to prevent cognitive deficits and symptoms of depression during alcohol detoxification. This trial used a double-blind placebo-controlled design.

### Future directions

Several preclinical reports have shown that type II receptor glucocorticoid antagonists could be of value during alcohol detoxification. Since the current trial was started, Vendruscolo et al. [49] published results from a double-blind laboratory study in alcohol-dependent volunteers. Compared with placebo, mifepristone reduced alcohol-cued craving that has been shown to be predictive of relapse drinking and reduced self-reported alcohol consumption.

Mifepristone, in addition to its antagonism at type II glucocorticoid receptors, has progesterone antagonist properties. In the present study, the latter effect meant that the participants had all to be males. However, in recent years, type II receptor antagonists have been developed that do not possess any progesterone antagonism; ORG34517 is showing promising results in alcohol dependence.

Recruitment for this trial relied on the provision of NHS addiction treatment services with potential participants requiring 2 weeks of inpatient treatment to monitor administration of mifepristone. Due to the changes in treatment provision for alcohol addiction, recruitment of suitable participants was extremely problematic, with few patients receiving NHS inpatient treatment. Future research could develop a trial protocol that allows for patients receiving treatment as an outpatient. Careful consideration would need to be given to ensure the safety of patients is adequately monitored and adherence to the medication is maximised. The use of Clinical Research Facilities (CRFs) would provide an alternative strategy. CRFs can provide a dedicated space with specialist staff for clinical research. The costs for the use of such facilities can be considerable so would need to be considered when applying for research funding.

## Conclusion

This research was not able to determine the potential efficacy of glucocorticoid receptor antagonism for preventing cognitive deficits and symptoms of depression following an alcohol detox. Further research is warranted, but careful consideration needs to be given to research protocols that include participants completing an alcohol detoxification as an outpatient or the use of a CRF to maximise participant recruitment.

## Supplementary information

**Additional file 1: Supplementary table 1.** The profile of mood states (POMS), Snaith-Hamilton Pleasure Scale (SHAPS), Alcohol Urge Questionnaire (AUQ) and sleep quality at days 7, 14, 21 and 28 post randomisation to placebo or mifepristone.

**Additional file 2: Supplementary table 2.** The profile of mood states (POMS), Snaith-Hamilton Pleasure Scale (SHAPS), Alcohol Urge Questionnaire (AUQ) and sleep quality at 3, 6 and 12-months post randomisation to placebo or mifepristone.

## Data Availability

The datasets used and/or analysed during the current study are available from the corresponding author on reasonable request.

## Abbreviations

ALT: Alanine aminotransferase
AUQ: Alcohol Urge Questionnaire
BDI-II: Beck depression scale
CANTAB: Cambridge Neuropsychological Test Automated Battery
CIDI: Composite International Diagnostic Interview
POMS: Profile of Mood States
SADQ: Severity of Alcohol Dependence Questionnaire
SHAPS: Snaith-Hamilton Pleasure Scale
TLFB: Timeline Follow-Back
