# Supplementary material for: Double-blind, placebo-controlled trial of mifepristone on cognition and depression in alcohol dependence
Source: Trials. 2020 Sep 16;21:796. doi: 10.1186/s13063-020-04726-z (PMC7493392; doi:10.1186/s13063-020-04726-z)
Supplement: Supplementary file 1 — Additional file 1: Supplementary table 1. The profile of mood states (POMS), Snaith-Hamilton Pleasure Scale (SHAPS), Alcohol Urge Questionnaire (AUQ) and sleep quality at days 7, 14, 21 and 28 post randomisation to placebo or mifepristone. [file 13063_2020_4726_MOESM1_ESM.docx]

Supplementary table 1: The profile of mood states (POMS), Snaith-Hamilton Pleasure Scale (SHAPS), Alcohol Urge Questionnaire (AUQ) and sleep quality at days 7, 14, 21 and 28 post randomisation to placebo or mifepristone.

|  | Day 7 | | Day 14 | | Day 21 | | Day 28 | |
| --- | --- | --- | --- | --- | --- | --- | --- | --- |
|  | Placebo | Mifepristone | Placebo | Mifepristone | Placebo | Mifepristone | Placebo | Mifepristone |
| **POMS, median (IQR) [n]** | | | | | | | | |
| Anxiety | 1.56 (1) [11] | 1.44 (0.89) [13] | 1.33 (0.53) [10] | 1.33 (0.69) [10] | 1.44 (0.58) [10] | 1.33 (0.78) [11] | 1.28 (0.42) [10] | 1.57 (1) [11] |
| Depression | 1.27 (1) [11] | 1.07 (0.83) [13] | 1.17 (0.62) [10] | 0.97 (0.73) [10] | 1.23 (0.67) [10] | 1.20 (0.40) [11] | 0.97 (0.37) [10] | 0.93 (0.60) [11] |
| Anger | 1.92 (0.75) [11] | 1.50 (0.83) [13] | 1.29 (0.60) [10] | 1.38 (0.31) [10] | 1.50 (0.69) [10] | 1.50 (0.67) [11] | 1.38 (0.63) [10] | 1.50 (0.58) [11] |
| Vigour | 2.00 (0.88) [11] | 2.00 (0.63) [13] | 2.21 (0.50) [10] | 1.95 (0.88) [10] | 2.25 (0.88) [10] | 1.88 (0.75) [11] | 2.13 (0.66) [10] | 2.00 (0.75) [11] |
| Fatigue | 0.71 (1.43) [11] | 0.57 (1.29) [13] | 0.64 (0.75) [10] | 0.71 (1) [10] | 0.43 (0.57) [10] | 0.86 (1.14) [11] | 0.50 (0.36) [10] | 0.71 (0.71) [11] |
| Confusion | 1.86 (0.86) [11] | 1.71 (0.57) [13] | 1.78 (0.79) [10] | 1.43 (0.46) [10] | 1.71 (0.71) [9] | 1.71 (0.86) [10] | 1.71 (0.64) [10] | 1.71 (0.43) [10] |
| Friendship | 1.86 (1.14) [11] | 1.57 (1) [13] | 1.86 (1.29) [10] | 1.28 (0.89) [10] | 1.28 (0.79) [9] | 1.57 (0.57) [11] | 1.43 (0.75) [10] | 1.43 (1) [11] |
| Elation | 1.50 (1) [11] | 1.33 (0.92) [13] | 1.33 (0.89) [10] | 1.33 (0.71) [10] | 1.67 (0.58) [9] | 1.33 (0.67) [11] | 1.33 (0.58) [10] | 1.17 (1.17) [11] |
| Arousal | 1.14 (1.83) [11] | 0.98 (1.81) [13] | 1.02 (1.22) [10] | 1.30 (1.09) [10] | 1.13 (0.73) [9] | 1.00 (1.55) [11] | 1.13 (1.09) [10] | 1.16 (1.15) [11] |
| Positive | 0.03 (0.50) [11] | 0.37 (0.65) [13] | 0.13 (0.59) [10] | 0.25 (0.92) [10] | 0.13 (0.50) [9] | 0.27 (0.50) [11] | 0.32 (0.31) [10] | 0.23 (0.73) [11] |
| **SHAPS, n (%)** | | | | | | | | |
| Normal | 9 (81.8) | 11 (84.6) | 10 (90.0) | 10 (76.9) | 9 (81.8) | 10 (76.0) | 8 (72.7) | 12 (92.3) |
| Possible Anhedonia | 2 (18.2) | 2 (15.4) | 1 (9.9) | 3 (23.1) | 2 (18.2) | 3 (23.1) | 3 (27.3) | 1 (7.7) |
| **AUQ, median (IQR) [n]** | 14 (16) [11] | 15.5 (22) [12] | 8.5 (10.5) [10] | 9 (9) [10] | 10 (9.25) [10] | 9 (7) [11] | 8 (8.75) [10] | 14 (16) [11] |
| **Sleep, median (IQR) [n]** | 7 (4) [11] | 8 (5) [13] | 6.5 (2) [10] | 7 (5) [11] | 7 (2) [10] | 8 (4) [11] | 6 (3) [10] | 7 (3) [11] |
| **BDI, median (IQR) [n]** | 15.5 (19.75) [10] | 23 (23) [13] | 10 (15.5) [10] | 15.3 (22.75) [8] | 8.5 (16) [10] | 13.5 (16.5) [10] | 7 (11.75) [10] | 15.5 (12.5) [10] |

NB: POMS = Profile of Mood States, SHAPS = Snaith-Hamilton Pleasure Scale, AUQ = Alcohol Urge Questionnaire, BDI = Beck Depression Inventory-II
