# Supplementary material for: Double-blind, placebo-controlled trial of mifepristone on cognition and depression in alcohol dependence
Source: Trials. 2020 Sep 16;21:796. doi: 10.1186/s13063-020-04726-z (PMC7493392; doi:10.1186/s13063-020-04726-z)
Supplement: Supplementary file 2 — Additional file 2: Supplementary table 2. The profile of mood states (POMS), Snaith-Hamilton Pleasure Scale (SHAPS), Alcohol Urge Questionnaire (AUQ) and sleep quality at 3, 6 and 12-months post randomisation to placebo or mifepristone. [file 13063_2020_4726_MOESM2_ESM.docx]

Supplementary table 2: The profile of mood states (POMS), Snaith-Hamilton Pleasure Scale (SHAPS), Alcohol Urge Questionnaire (AUQ) and sleep quality at 3, 6 and 12-months post randomisation to placebo or mifepristone.

|  | 3-month follow-up | | 6-month follow-up | | 12-month follow-up | |
| --- | --- | --- | --- | --- | --- | --- |
|  | Placebo, n = 9 | Mifepristone, n = 7 | Placebo, n = 8 | Mifepristone, n = 5 | Placebo, n = 8 | Mifepristone, n = 2 |
| **POMS, Median (IQR) [n]** | | | | | | |
| Anxiety | 1.33 (0.61) [9] | 1.67 (1.11) [7] | 1.11 (0.19) [8] | 2.11 (1) [5] | 1.28 (0.22) [8] | 1.50 (na) [2] |
| Depression | 1.07 (0.73) [9] | 1.53 (1.07) [7] | 0.77 (0.25) [8] | 1.93 (1.33) [5] | 0.93 (0.68) [8] | 1.73 (na) [2] |
| Anger | 1.42 (0.33) [9] | 1.42 (1.33) [7] | 1.17 (0.4) [8] | 1.67 (1.83) [5] | 1.42 (0.31) [8] | 1.75 (na) [2] |
| Vigour | 2 (0.88) [9] | 1.50 (1.13) [7] | 2 (0.88) [8] | 1.88 (1.56) [5] | 2.06 (0.81) [8] | 2.25 (na) [2] |
| Fatigue | 0.57 (0.86) [9] | 1.28 (1.29) [7] | 0.43 (0.75) [8] | 1.86 (2.07) [5] | 0.50 (1.36) [8] | 1.28 (na) [2] |
| Confusion | 1.43 (0.36) [9] | 1.57 (0.57) [7] | 1.57 (0.86) [8] | 1.86 (1.14) [5] | 1.43 (0.71) [8] | [1] |
| Friendship | 1.14 (0.64) [9] | 1.43 (1.29) [7] | 1 (0.61) [8] | 2 (1.50) [5] | 1.36 (0.39) [8] | 1.92 (na) [2] |
| Elation | 1 (0.58) [9] | 1.50 (1.33) [7] | 1 (0.42) [8] | 2.17 (1.50) [5] | 1.17 (0.79) [8] | 1.92 (na) [2] |
| Arousal | 1.43 (1.06) [9] | 0.44 (0.97) [7] | 1.13 (1.14) [8] | 0.54 (3.16) [5] | 1.33 (1.85) [8] | 0.75 (na) [2] |
| Positive | 0.20 (0.97) [9] | -0.07 (0.97) [7] | 0.28 (0.27) [8] | 0.47 (1.15) [5] | 0.12 (0.35) [8] | 0.18 (na) [2] |
| **SHAPS, N (%)** | | | | | | |
| Normal | 8 (72.7) | 11 (84.6) | 7 (63.6) | 13 (100) | na | na |
| Possible Anhedonia | 3 (27.3) | 2 (15.4) | 4 (36.4) | 0 (0) | na | na |
| **AUQ, median (IQR) [n]** | 8 (8) [9] | 22 (24) [7] | 8.5 (9.75) [8] | 33 (33.5) [5] | 8 (1) [7] | 24.5 (na) [2] |
| **Sleep, median (IQR) [n]** | 6 (3) [9] | 6 (6) [7] | 7 (3) [8] | 5 (4) [5) | 7.5 (6) [8] | 3.5 (na) [2] |
| **BDI, median (IQR) [n]** | 4 (9.5) [8] | 22 (16) [7] | 3.5 (14.75) [8] | 24 (24.5) [5] | 1 (24.5) [8] | 13.5 (na) [2] |

NB: POMS = Profile of Mood States, SHAPS = Snaith-Hamilton Pleasure Scale, AUQ = Alcohol Urge Questionnaire, BDI = Beck Depression Inventory-II
